# Financial burden of survivors of medically-managed myocardial infarction and its association with selected social determinants and quality of life in a lower middle income country

**DOI:** 10.1186/s12872-017-0687-y

**Published:** 2017-09-19

**Authors:** P. K. B. Mahesh, M. W. Gunathunga, S. Jayasinghe, S. M. Arnold, D. S. V. Mallawarachchi, S. K. Perera, U. A. D. Wijesinghe

**Affiliations:** 1 Office of Regional Director of Health Services, Colombo, Sri Lanka; 2 Department of Community Medicine, Faculty of Medicine, University of Colombo, Colombo, Sri Lanka; 3 Department of Clinical Medicine, Faculty of Medicine, University of Colombo, Colombo, Sri Lanka; 4 Non Communicable Disease Unit, Ministry of Health, Colombo, Sri Lanka; 5 Post Graduate Institute of Medicine, University of Colombo, Colombo, Sri Lanka; 6 Cardiology Unit, National Hospital Sri Lanka, Colombo, Sri Lanka

**Keywords:** Myocardial infarction, financial burden, Quality of life, Social Determinants of Health, EQ-5D-3 L, STEMI, NSTEMI

## Abstract

**Background:**

Burden from ischemic heart disease is rising in Sri Lanka due to the demographic and epidemiological transitions**.** Documented literature is scarce on quality of life, financial burden and its determinants in relation to myocardial infarction (MI). This study was done to describe the financial burden among the survivors of MI managed only with drugs (i.e. those who did not undergo Percutaneous Coronary Intervention or Coronary Artery Bypass Graft) and its association with selected social determinants (SDHs) and quality of life (QOL).

**Methods:**

A cross sectional study was done among MI survivors in 13 hospitals in the premier province of Sri Lanka. Out of 336 participants recruited at hospital stay, 270 responded through a self-administered questionnaire at 1 month post discharge. Questionnaire included sections on financial burden, selected SDHs and on QOL measured by the EQ-5D-3 L QOL tool. Presence of financial burden was determined using an operational definition. Associations were tested with Mann–Whitney-U test, Chi square test and Spearman-correlation-coefficient at 5% significant level.

**Results:**

Around 40% (*n* = 116) had to seek financial support for out-of-pocket expenditure. Nearly 5% (*n* = 6) of previously employed participants had lost their job. Of the employed respondents (*n* = 139, 51.5%)**,** 29% (*n* = 85) had limited physical activity and 40% (*n* = 115) had limitations of employment time. Of the respondents, 15.4% had to apply for a loan, 7.8% had to sell a property, 19.1% had an income loss and 33.8% had to restrict usual expenses. Financial burden was not significantly associated with gender (*p* = 0.146), ethnicity (*p* = 0.068), highest education (*p* = 0.184) and area of residence (*p* = 0.369). Influence of income (*p* = 0.001), social support (*p* = 0.002) and the health infrastructure (*p* < 0.001) were significantly associated with the occurrence of a financial burden. In the group with a financial burden, the index score (p = 0.002) and VAS score (p < 0.001) of EQ-5D-3 L were significantly lower.

**Conclusions:**

Financial burden is common among survivors of medically-managed occurring irrespective of the gender, ethnicity, education and the area. It is influenced significantly by the income, level of social support and the level of health infrastructure. The financial burden is influencing the post-discharge-1-month QOL.

## Background

Chronic Non Communicable Diseases (NCDs) impose a severe threat for lower and middle income countries with Sri Lanka being a no exception [1–3]. Cardiovascular diseases are a category with high global disease burden falling under the chronic NCDs [4–6]. Among cardiovascular diseases, myocardial infarction (MI) is a main acute critical events endangering the lives of affected people and decreasing the quality of life [7–9].

It has been shown that chronic NCDs potentially make all people vulnerable to a financial risk and this risk is more in poorer people [2, 10–12]. The magnitude of Catastrophic Health Expenditure (CHE) is significantly associated with many social factors [13, 14]. It is acknowledged that health cannot be improved without addressing the Social Determinants of Health (SDHs) [15]. The WHO Commission on Social Determinants of Health (CSDH) has introduced the CSDH conceptual framework for action on SDHs [14, 16].

Literature from the South Asian region shows that, in some occasions a significant amount of total household expenditure is spent for healthcare payments [3, 10, 17]. Making the situation worse, when a critical event is experienced on top of chronic NCDs, more financial burden is resulted. In relation to critical illnesses, the similar findings have been highlighted in the global literature [18–22].

In MI, patients are managed as in-ward patients and are later followed up at out-patient ambulatory clinics. Even though the state services provide free-of-charge care in Sri Lanka, there are instances patients have to experience out-of-pocket expenditure and indirect costs [2]. When concomitant comorbid conditions are found the financial burden for concomitant coronary heart disease is even higher [13].

Exploring the financial burden of the survivors of MI provides the true scenario on aspects like equity in healthcare. The evaluation of the associations between SDHs with the financial burden, provide scientific hints for the prioritization of families that need potential financial support. Furthermore the evaluation of the association of the financial burden with QOL would reveal the impact of the disease on their lifestyle as a whole. In relation to the association between financial burden and social determinants, comprehensive literature is not commonly found globally [23]. In relation to the lower middle income countries like Sri Lanka, the documented literature on these aspects is rarer. This study was done to bridge this gap with the aim of describing the financial burden faced by the survivors of MI who were medically managed [i.e. without Percutaneous Coronary Intervention (PCI) or Coronary Artery Bypass Graft (CABG)] and its association with selected social determinants and quality of life.

## Methods

The study included two cross sectional data collections in 13 government hospitals, in the Western Province of Sri Lanka. Study population included the patients who were admitted to the above settings with the diagnosis of MI. Inclusion criteria included the criteria for the diagnosis of MI Category A of the 2008–2009 revisions of the World Health Organization’s diagnostic criteria [24]. These included: at least one value of increased level of cardiac biomarkers (preferably troponin) with at least one of the following; development of pathological Q waves, symptoms of ischemia, ECG changes (new ST-T changes or new left bundle branch block) and imaging evidence [24]. The demarcation of STEMI and NSTEMI were done based on European Society for Cardiology guidelines [25]. Participants with duration of stay less than 48 h, participants who underwent invasive interventions (i.e. PCI or CABG) and with known pre-MI physical or mental disabilities which would potentially reduce the quality of life were excluded.

All patients who satisfy the above criteria who were admitted to the mentioned 13 settings within the Western province were selected as eligible for the study. Sample size was calculated with the formula n = z^2^ x p (1-p)^2^/d^2^ getting the expected prevalence of financial burden based on documented literature [20, 21, 23, 26]. It was calculated that a minimal number of 235 records would be needed at data analysis stage. With an assumed response rate of 70%, the minimum sample size needed to be recruited at the data collection stage was 336. Data collection started simultaneously in all the study settings on 1st of January 2015 and continued until the sample size was in March 2015.

Data collection was done by the investigators and seven trained data collectors who were medically qualified graduates prior to their medical internship. Data collectors daily visited the medical wards that were respectively allocated to them out of the study settings. They verified the inclusion criteria of the patients who had been diagnosed with MI by the ward staff. A self-administered questionnaire was given to the participants with a stamped envelope addressed to the principal investigator. The contact details were obtained and a reminder was given at the completion of 28 days.

The questionnaire consisted of three sections; data on the post-discharge 1 month QOL, financial burden and the social determinants. The post-discharge 1-month QOL was assessed using the EQ-5D-3 L generic QOL tool. The financial burden section included the questions formed with the literature review and expert guidance. The questions were based on the operational definition of “a situation in which the participant has experienced an actual or potential financial loss and is worried about it”. Four structural SDHs (i.e. gender, ethnicity, income and highest education level) and three intermediate SDHs (place of living, social support and level of health infrastructure) were selected as contextually relevant and as influencing the financial burden [15, 16].

Gender, Ethnicity and residence were coded as categorical variables. Place of living was categorized as “urban” or “rural” according to the “concepts and definitions” of the department of census and statistics [27]. Income, highest educational level, level of social support and health infrastructure were noted as quantitative variables. For measuring the social support a modified tool was used with a maximum score of 10, based on the set of questions used by Mallawarachchi in 2007 (Table 1) [28, 29].

**Table 1:** Scoring method of the tool to assess the level of social support

Question	Score
Availability of someone to call	1
Availability of someone to talk to	1
Availability of someone to encourage	1
Availability of someone to help in daily activities	1
Availability of someone to help in house keeping	1
Availability of someone to take to the doctor	1
Availability of someone to provide financial assistance	1
Availability of someone to provide a vehicle	1
Availability of someone to give information about the disease	1
Availability of help from any institution/ group	1
Total	10

Following literature review and expert opinions, a judgmentally validated tool was used for quantifying the level of health infrastructure with a maximum score of 5 (Table 2) [29]. The consulted expert panel included local specialists from the fields of medicine, primary-care and community medicine.

**Table 2:** Scoring method for the tool for quantifying the health infrastructure

Domain	Scores and criteria	
i. Distance to the nearest pharmacy	< 1km 0.5	> 1 km 0
ii. Distance to the nearest place a doctor can be consulted	< 1km 1	> 1 km 0
iii. Distance to the nearest hospital	< 1km 1	> 1 km 0
iv. Distance to the nearest hospital with specialist care	< 1km 1	> 1 km 0
v. Availability of a healthcare personnel for health advices/ clarifications	Yes 1	No 0
vi. Ownership of vehicles	Yes 0.25	No 0
vii. Condition of roads	Does not cause delays 0.25	Cause delays 0

The severity of illness of MI was described with the Thrombolysis in Myocardial Infarction (TIMI) risk score [30]. For NSTEMI it was calculated by allocating a score out of seven; four of which was related to the past medical history and three for the clinical presentation [31]. For STEMI, the score was out of maximum 14; four of which was given for the past medical history, eight for the examinations and two for presentation [32]. The higher the TIMI score, the worse the severity of the disease.

Data was entered into a pre-designed sheet in Statistical package for Social Sciences (SPSS version 20). Demographic factors and several aspects of financial burden were elaborated using descriptive statistics. The group with financial burden was defined as at least having one of the phenomena mentioned in Table 3. The association between the presence of financial burden and the SDHS in categorical form was evaluated by the chi square test. The score of the SDHs which were in quantitative form and the QOL scores were non-normally distributed when assessed by Q-Q plots and Kolmogorov–Smirnov test. Hence non-parametric tests were used in analyzing their associations. Mann Whitney U test was used for analysis of associations of financial burden with numerically-measured SDHs and with QOL. The same test was used to analyze for the association between QOL and the type of MI. Spearman correlation coefficient was used to describe the association between QOL versus TIMI risk score and left ventricular ejection fraction prior to hospital discharge.

**Table 3:** Financial burden experienced by the study population

Burden	Yes	No	Not mentioned
	N (%)	N (%)	N (%)
Had to apply for a loan	45 (15.4)	209 (71.3)	16 (13.3)
Had to sell a property	23 (7.8)	222 (75.8)	25 (16.4)
Had a potential income loss	56 (19.1)	192 (65.5)	22 (15.4)
Had to restricted other expenses	99 (33.8)	154 (52.6)	17 (13.6)

Informed written consent was obtained similar to methods used by Hofhuis et al. in 2007 [33]. Data collections were done without disturbing the patient management processes. Ethical clearance was obtained and administrative permissions were obtained prior to the initiation of data collection.

## Results

The median (IQR) of the age distribution of the recruited study sample was 62.0 (54.0–70.0). Out of the recruited participants 270 either posted back the questionnaire or gave the responses over the phone with a general response rate of 80.8%. The non-responders had either died following the hospital admission or could not be contacted. The analysis of the characteristics of responders and non-responders showed that the non-response bias is unlikely [34, 35].

Out of the respondents 65 were with a STEMI and 205 were with NSTEMI. The median (IQR) age was 58 (51–67) years. Male to female ratio was 7:3with 186 males and 84 females. The median (IQR) TIMI score for the STEMI group was 3 (3 to 5) and for NSTEMI was 2 (2 to 4). All recruited STEMI patients were given streptokinase as the intravenous fibrinolytic therapy and NSTEMI patients received parenteral anticoagulant therapy.

Out of the responders, 139 (51.5%) had been employed by 1 month of hospital discharge. Around 31% (*n* = 85) of the employees stated that they had a limitation of physical activity and around 43% (*n* = 115) had to limit the employment time due to the effects of the MI. Nearly 5% (*n* = 6) of those who were not employed by one month had lost their previously engaged job due to the illness.

Around 43% (*n* = 116) have stated that they had to request the financial support from a third party due to the illness. Around 86% (*n* = 231) need to have someone to accompany him or her for any form of further medical management following hospital discharge. Out of these companions, around 60% are engaged in a day-time job and they had to limit some portion of their working time for accompanying the participant.

Table 3 summarizes the financial burden experienced by the participants in relation to four selected questions. Out of the respondents, 137 (50.7%) had experienced at least one of the four occurrences mentioned.

The Table 4 shows that the group without financial burden has better figures for the education level, income, level of social support and the level of health infrastructure. When analyzed by the non-parametric Mann Whitney U test the latter three show a statistically significant difference between the two groups.

**Table 4:** Association between financial burden and selected social determinants

Social determinant	Financial burden category	Mean (SD)	Median (IQR)	Significance of difference^a^
Education level	With burden	8.09 (3.91)	9.00 (5.00–11.00)	*p* = 0.184
	No burden	8.65 (4.15)	10.0 (5.50–11.00)	
Income	With burden	2.20 (1.32)	2.00 (1.00–3.00)	*p* = 0.001^b^
	No burden	2.69 (1.31)	3.00 (2.00–4.00)	
Social support	With burden	7.03 (2.13)	7.00 (6.00–9.00)	*p* = 0.002^b^
	No burden	7.83 (1.79)	8.00 (7.00–9.00)	
Health infrastructure	With burden	1.86 (1.26)	1.50 (0.75–2.75)	*p* < 0.001^b^
	No burden	3.28 (1.04)	3.25 (2.50–4.13)	

^a^Analyzed by the Mann–Whitney U test

^b^significant at 5% level

The SDHs mentioned in Table 5 which are gender, ethnicity and place of living do not show a statistically significant different association between the two groups.

**Table 5:** Association between financial burden and selected social determinants

Selected SDH	Yes	No	Total	Significance of difference^a^
	N (%)	N (%)	N (%)	
Gender				
-Male	98	85	183 (100)	*p* value = 0.146
-Female	36	46	82 (100)	
Ethnicity				
-Sinhala	117	100	217 (100)	*p* value = 0.068
-Others	18	28	46 (100)	
Area				
-Urban	79	70	149 (100)	*p* value = 0.369
-Rural	35	40	75 (100)	

^a^Analyzed by the chi-square test

The QOL at post-discharge 1 month, shows a significant difference between the two groups in relation to both the index value (*p* = 0.002) and the Visual Analogue Scale (VAS) value (*p* < 0.001) (Table 6) of the EQ-5D-3 L QOL tool.

**Table 6:** Association between financial burden and the quality of life

QOL	Financial burden category	Mean (SD)	Median (IQR)	Significance of difference^a^
EQ-5D index value	With burden	0.486 (0.38)	0.516 (0.49–0.73)	*p* = 0.002^b^
	No burden	0.671 (0.19)	0.585 (0.52–0.85)	
EQ-5D VAS value	With burden	60.97 (21.46)	60.0 (50.00–77.50)	*p* < 0.001^b^
	No burden	71.56 (15.39)	70.0 (60.00–80.00)	

^a^Analyzed by Mann Whiney U test

^b^significant at 5% level

EQ-5D index value did not show a significant association with the TIMI risk score for the STEMI group (*p* = 0.075) nor with the NSTEMI group (*p* = 0.058). Similarly the EQ-5D VAS values too, did not show a statistically significant association between STEMI (*p* = 0.35) or NSTEMI groups (*p* = 0.364). Furthermore in a subgroup analysis of approximately one fourth of the participants, the left ventricular ejection fraction prior to hospital discharge did not show a significant association with either the EQ-5D-index score (*p* = 0.222) or the EQ-5D-VAS score (*p* = 0.693). Neither the EQ-5D index score (*p* = 0.392) nor the EQ-5D-VAS (*p* = 0.125) showed a statistically significant difference between STEMI and NSTEMI groups.

## Discussion

The study shows that a substantial number of MI survivors had experienced a financial burden. Furthermore, the financial burden experienced by them has a significant association with three SDHs and with the post-discharge QOL at one month. The Sri Lankan health financing system involves two main sources: general taxation and out-of-pocket household spending [2]. Private insurance is a supplementary-optional method. Since the uptake of insurance is weak, there is a potential for out-of-pocket expenditures to be incurred by patients with MI. Higher magnitude of Out of Pocket expenditure is a major restriction in the country’s path towards achieving Universal Health Coverage. It not only leads to impoverishment, but also restricts access to medical care.

The financial burden summarized in Table 3 needs to be interpreted while recognizing the presence of co-morbid conditions. Some participants, who were previously unaware about the presence of co-morbidities, had them detected during the current admission. Managing these co-morbidities would have worsened the financial burden. For example if a drug becomes temporarily out-of-stock in the hospital pharmacy, the patient would need to purchase it from a private pharmacy.

Following one month of the hospital discharge, 51.5% mentioned that they were working. A quicker return to work may have been necessitated by the economic burden as documented in global literature too [18]. The employed participants had to face some limitations in activities and had to compromise some aspects of the lifestyle comparable to the global literature [23]. The financial restraints are major concerns of the survivors of chronic illnesses as documented in the global literature [18, 20, 21, 23, 36]. Similarly the present study shows that in a lower-middle-income country these concerns remain the same. These results indicate that the economic consequences are multi-faceted and have a negative impact on the lifestyles. These issues are not usually captured by the routine health information systems.

The associations between SDHs in Tables 5 and 6 suggest that the gender (*p* = 0.146), ethnicity (*p* = 0.068), highest education (*p* = 0.184) and the area of residence (*p* = 0.369) are not significantly associated with the financial burden. Hence the findings can be generalized irrespective of the gender, ethnicity or place of living. The influence of income (*p* = 0.001), social support (*p* = 0.002) and the health infrastructure (*p* < 0.001) were noted as statistically significant. This fact strengthens the available scientific literature which shows the association between financial burden versus the SDHs [13, 14, 16]. When these SDHs were unfavorable, the financial burden was seemingly worse.

When the pre-event income is low, the presence of a financial burden following the MI is more likely. This would have been resulted by the lack of savings. A better level of social support minimizes the risk of the occurrence of financial burden. This may not be only due to the mere financial inputs and would be due to the other factors like knowledge sharing as well. Similarly a better health infrastructure would have a protective effect preventing the MI survivors going into financial burden.

The EQ-5D-3 L QOL tool has been developed by EuroQol group. It consist of EQ-5D descriptive system as well as EQ-5D VAS [37, 38]. It focuses on the day of the measurement of the QOL. It has been validated in previous studies in Sri Lanka [29, 39]. The minimum time taken for the completion and the relative simplicity make it a suitable QOL tool to be used to measure the one month post-hospital discharge QOL of MI survivors. The results revealed a significantly lower QOL in relation to both components (index score and VAS) among the group which was with a financial burden (Table 6). It points towards the importance of financial interventions in improving the QOL of MI survivors.

MI has significant negative consequences for the sufferers in relation to physical, emotional and social dimensions. Globally it is shown that the QOL of the coronary patients anyhow is significantly impaired following the illness [7, 40–42]. Measuring QOL of cardiac patients provide the opportunity of reflecting the success of the management in the patient’s point of view [43].

This is an era that the whole world is concerned about the equity issues in relation to health financing. Different income inequality measures are available in the literature such as; Gini coefficient, Atkinson index, generalized-entrophy index, Kakwani index [44–46]. It is timely that lower-middle-income countries like Sri Lanka encourage more scientific literature on these regards. This further provides evidence on actual necessity of health-economic reforms like social insurance systems and guided donations.

There were several limitations of the study. The financial burden was in categorical form and the quantification with a monetary value was not done. It was the best possible option as exploring quantification evidence could not be possible especially when the respondent is not exposed to a face-to-face interview. Secondly the out-of-pocket expenditure in some patients may be due to the temporary scarcities of certain medicines in particular hospitals requiring the patient to purchase them. This should be considered in interpreting the results. Thirdly the present study only recruited the medically-managed survivors of MI. This was due to the fact that at the time of data collection, there was no uniform financial-supporting system for all the MI patients who were managed with invasive interventions. Hence the exclusion of these patients enabled the applicability of the findings for the medically-managed patients in relation to the financial-burden, even if the policies related to surgical interventions change from time to time. However, since the mode of management may influence the post-discharge QOL, the findings of the present study on the QOL too, should not be generalized to the survivors of MI who underwent an invasive intervention. Since the study did not have an objective of describing any incidence-related parameter, the total participants who were screened for eligibility was not documented. Finally the left ventricular ejection fraction analysis was done for only one fourth of the respondents. This should be concerned in interpreting its associations.

## Conclusions

The survivors of MI who are medically-managed, have to face financial burden due to the out-of-pocket expenditure as well as due to the limitations the illness imposes on their occupations. This burden occurs irrespective of the gender, ethnicity, education and the place of residence. It is influenced significantly by the income, level of social support and the level of health infrastructure. The financial burden is influencing the post-discharge 1 month QOL of the medically-managed MI survivors. More research should be encouraged in these regards and the evidences must be coupled with health reforms to mitigate the impact of financial burden due to illness.

## Abbreviations

CABG: Coronary Artery Bypass Graft
CHE: Catastrophic Health Expenditure
CSDH: Commission on Social Determinants of Health
EQ-5D-3 L: EuroQol-five-dimensional-3-level questionnaire
MI: myocardial infarction
NCDs: Non Communicable Diseases
NSTEMI: Non- ST elevation myocardial infarction
PCI: Percutaneous Coronary Intervention
QOL: quality of life
SDHs: social determinants
STEMI: ST elevation myocardial infarction
TIMI risk score: Thrombolysis in Myocardial Infarction (TIMI) risk score
VAS: Visual Analogue Scale
